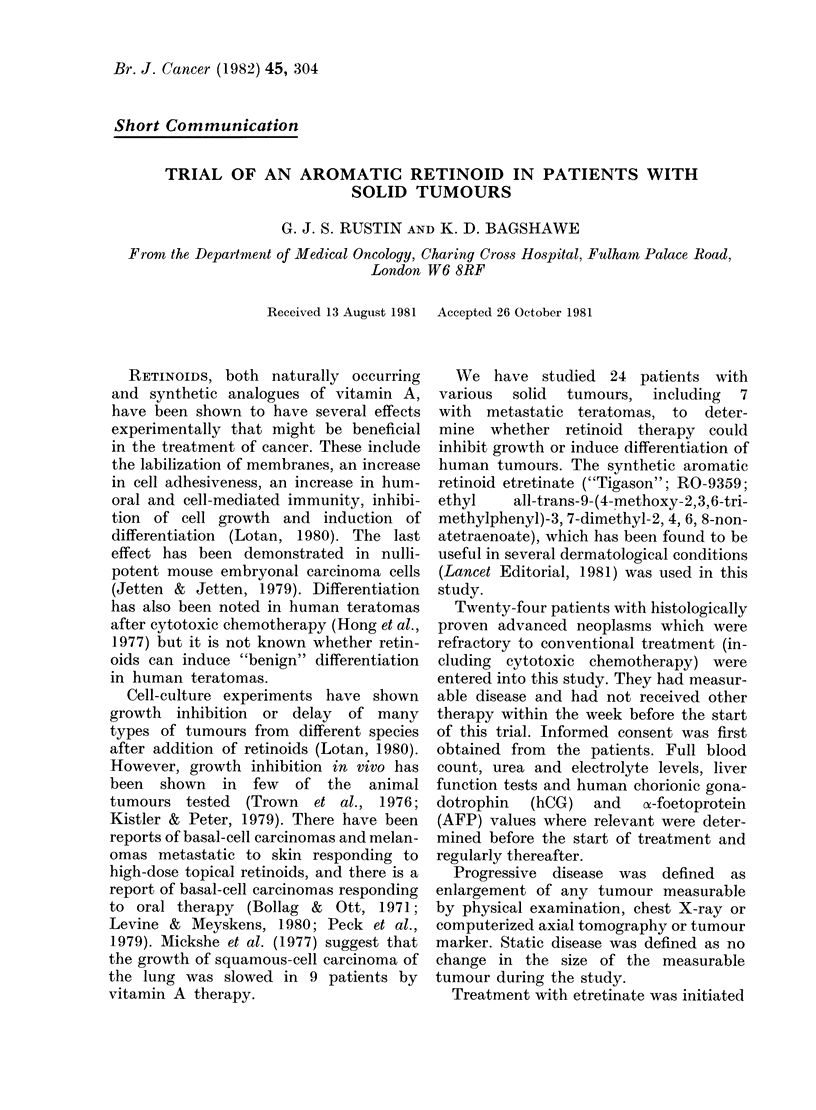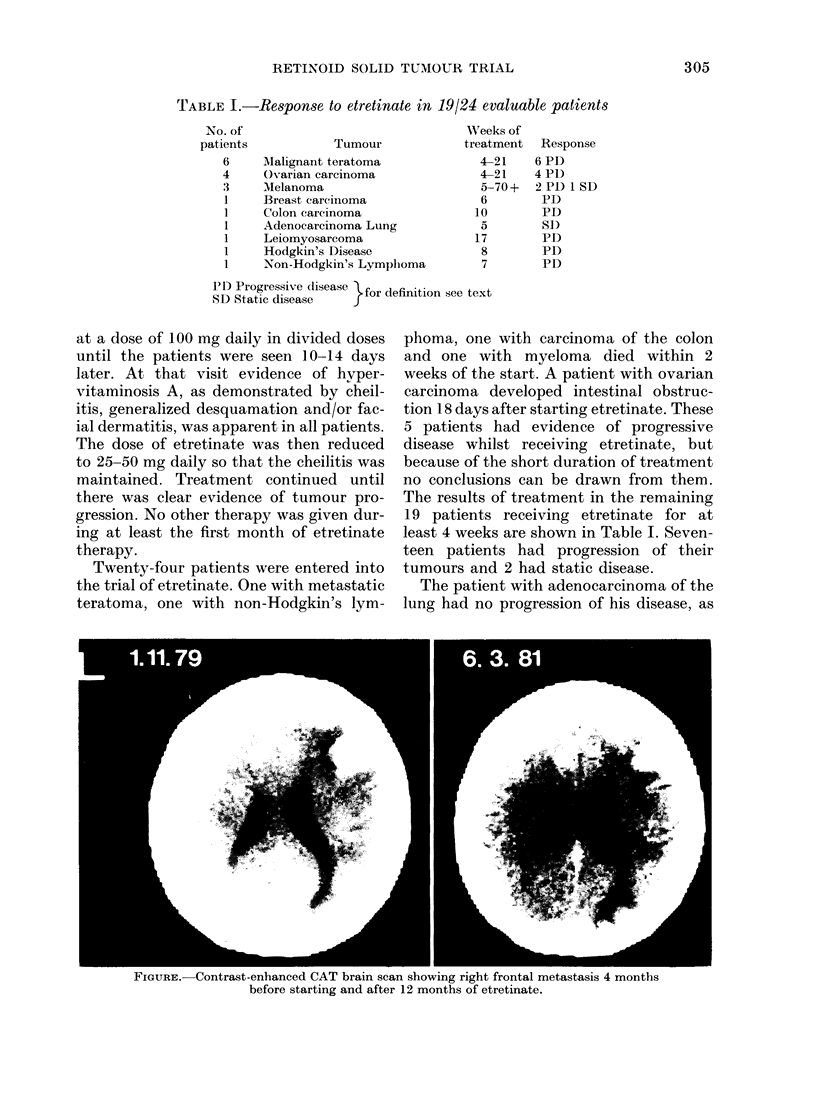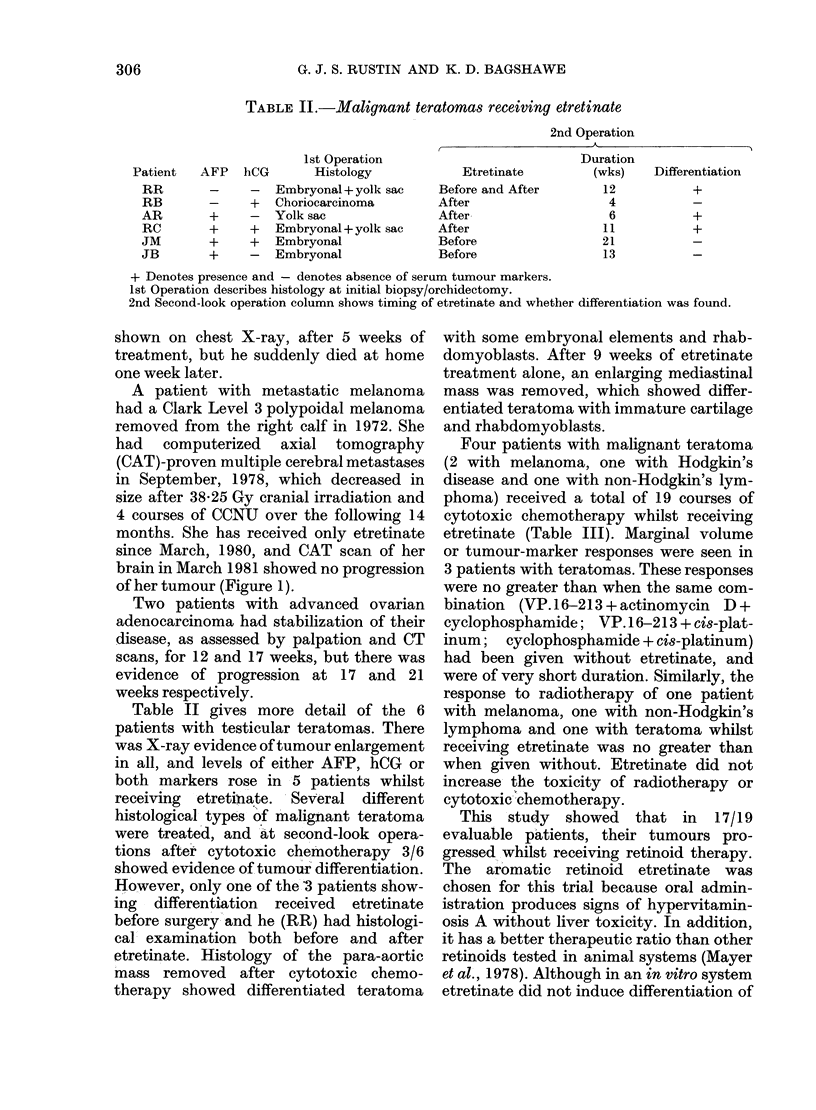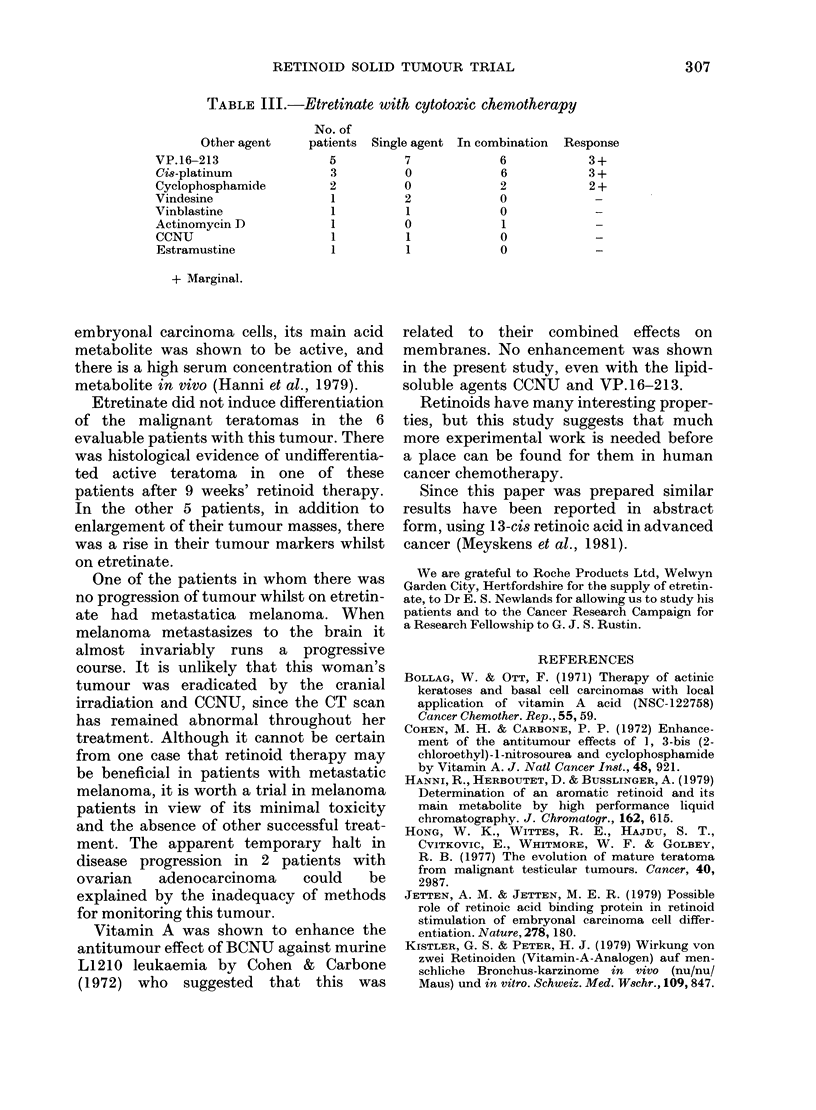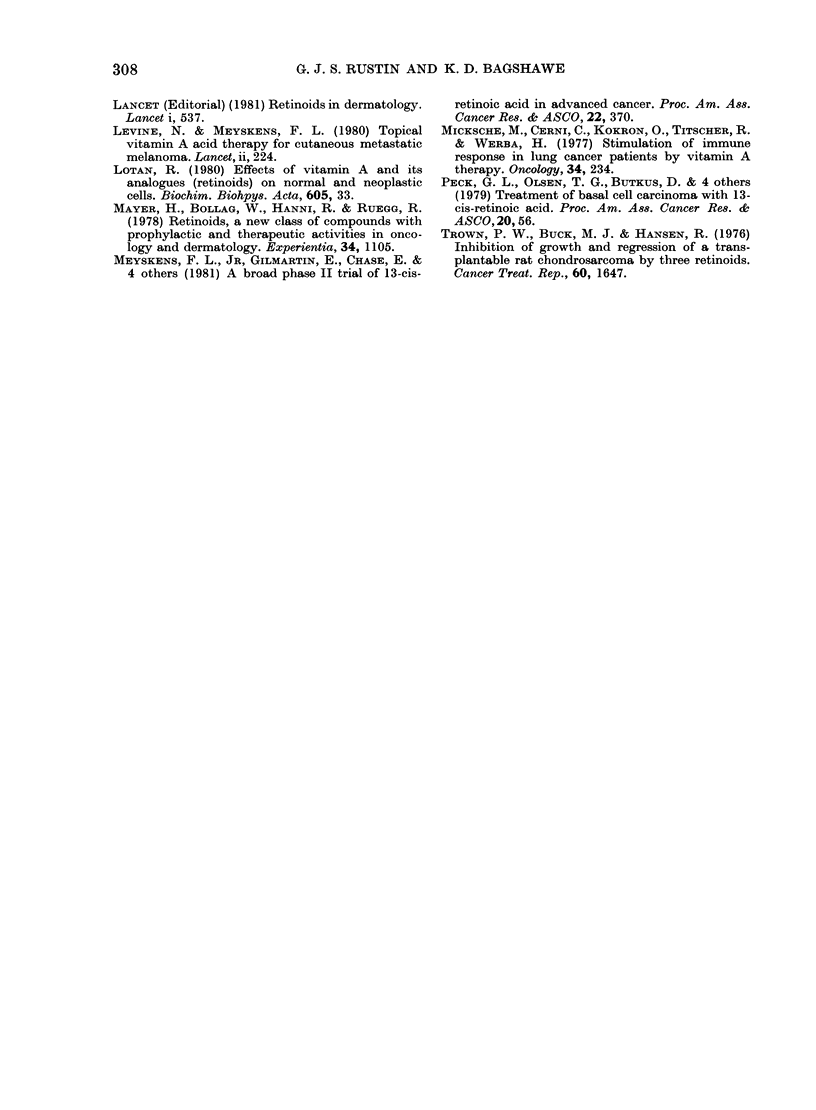# Trial of an aromatic retinoid in patients with solid tumours.

**DOI:** 10.1038/bjc.1982.48

**Published:** 1982-02

**Authors:** G. J. Rustin, K. D. Bagshawe

## Abstract

**Images:**


*Br. J. Cancer* (1982) **45**, 304

***Short Communication***

### TRIAL OF AN AROMATIC RETINOID IN PATIENTS WITH SOLID TUMOURS

G. J. S. RUSTIN AND K. D. BAGSHAWE

*From the Department of Medical Oncology, Charing Cross Hospital, Fulham Palace Road, London W6 8RF*

Received 13 August 1981 Accepted 26 October 1981

RETINOIDS, both naturally occurring and synthetic analogues of vitamin A, have been shown to have several effects experimentally that might be beneficial in the treatment of cancer. These include the labilization of membranes, an increase in cell adhesiveness, an increase in humoral and cell-mediated immunity, inhibition of cell growth and induction of differentiation (Lotan, 1980). The last effect has been demonstrated in nullipotent mouse embryonal carcinoma cells (Jetten & Jetten, 1979). Differentiation has also been noted in human teratomas after cytotoxic chemotherapy (Hong *et al.*, 1977) but it is not known whether retinoids can induce "benign" differentiation in human teratomas.

Cell-culture experiments have shown growth inhibition or delay of many types of tumours from different species after addition of retinoids (Lotan, 1980). However, growth inhibition *in vivo* has been shown in few of the animal tumours tested (Trown *et al.*, 1976; Kistler & Peter, 1979). There have been reports of basal-cell carcinomas and melanomas metastatic to skin responding to high-dose topical retinoids, and there is a report of basal-cell carcinomas responding to oral therapy (Bollag & Ott, 1971; Levine & Meyskens, 1980; Peck *et al.*, 1979). Mickshe *et al.* (1977) suggest that the growth of squamous-cell carcinoma of the lung was slowed in 9 patients by vitamin A therapy.

We have studied 24 patients with various solid tumours, including 7 with metastatic teratomas, to determine whether retinoid therapy could inhibit growth or induce differentiation of human tumours. The synthetic aromatic retinoid etretinate ("Tigason"; RO-9359; ethyl all-trans-9-(4-methoxy-2,3,6-trimethylphenyl)-3, 7-dimethyl-2, 4, 6, 8-nonatetraenoate), which has been found to be useful in several dermatological conditions (*Lancet* Editorial, 1981) was used in this study.

Twenty-four patients with histologically proven advanced neoplasms which were refractory to conventional treatment (including cytotoxic chemotherapy) were entered into this study. They had measurable disease and had not received other therapy within the week before the start of this trial. Informed consent was first obtained from the patients. Full blood count, urea and electrolyte levels, liver function tests and human chorionic gonadotrophin (hCG) and α-foetoprotein (AFP) values where relevant were determined before the start of treatment and regularly thereafter.

Progressive disease was defined as enlargement of any tumour measurable by physical examination, chest X-ray or computerized axial tomography or tumour marker. Static disease was defined as no change in the size of the measurable tumour during the study.

Treatment with etretinate was initiated

TABLE I.—*Response to etretinate in 19/24 evaluable patients*

| No. of patients | Tumour | Weeks of treatment | Response |
|---|---|---|---|
| 6 | Malignant teratoma | 4–21 | 6 PD |
| 4 | Ovarian carcinoma | 4–21 | 4 PD |
| 3 | Melanoma | 5–70+ | 2 PD 1 SD |
| 1 | Breast carcinoma | 6 | PD |
| 1 | Colon carcinoma | 10 | PD |
| 1 | Adenocarcinoma Lung | 5 | SD |
| 1 | Leiomyosarcoma | 17 | PD |
| 1 | Hodgkin's Disease | 8 | PD |
| 1 | Non-Hodgkin's Lymphoma | 7 | PD |

PD Progressive disease }

SD Static disease } for definition see text at a dose of 100 mg daily in divided doses until the patients were seen 10–14 days later. At that visit evidence of hypervitaminosis A, as demonstrated by cheilitis, generalized desquamation and/or facial dermatitis, was apparent in all patients. The dose of etretinate was then reduced to 25–50 mg daily so that the cheilitis was maintained. Treatment continued until there was clear evidence of tumour progression. No other therapy was given during at least the first month of etretinate therapy.

Twenty-four patients were entered into the trial of etretinate. One with metastatic teratoma, one with non-Hodgkin's lymphoma, one with carcinoma of the colon and one with myeloma died within 2 weeks of the start. A patient with ovarian carcinoma developed intestinal obstruction 18 days after starting etretinate. These 5 patients had evidence of progressive disease whilst receiving etretinate, but because of the short duration of treatment no conclusions can be drawn from them. The results of treatment in the remaining 19 patients receiving etretinate for at least 4 weeks are shown in Table I. Seventeen patients had progression of their tumours and 2 had static disease.

The patient with adenocarcinoma of the lung had no progression of his disease, as

FIGURE.—Contrast-enhanced CAT brain scan showing right frontal metastasis 4 months before starting and after 12 months of etretinate.

TABLE II.—*Malignant teratomas receiving etretinate*

| | | | | 2nd Operation | | |
|---|---|---|---|---|---|---|
| Patient | AFP | hCG | 1st Operation Histology | Etretinate | Duration (wks) | Differentiation |
| RR | − | − | Embryonal + yolk sac | Before and After | 12 | + |
| RB | − | + | Choriocarcinoma | After | 4 | − |
| AR | + | − | Yolk sac | After | 6 | + |
| RC | + | + | Embryonal + yolk sac | After | 11 | + |
| JM | + | + | Embryonal | Before | 21 | − |
| JB | + | − | Embryonal | Before | 13 | − |

+ Denotes presence and − denotes absence of serum tumour markers.
1st Operation describes histology at initial biopsy/orchidectomy.
2nd Second-look operation column shows timing of etretinate and whether differentiation was found.

shown on chest X-ray, after 5 weeks of treatment, but he suddenly died at home one week later.

A patient with metastatic melanoma had a Clark Level 3 polypoidal melanoma removed from the right calf in 1972. She had computerized axial tomography (CAT)-proven multiple cerebral metastases in September, 1978, which decreased in size after 38·25 Gy cranial irradiation and 4 courses of CCNU over the following 14 months. She has received only etretinate since March, 1980, and CAT scan of her brain in March 1981 showed no progression of her tumour (Figure 1).

Two patients with advanced ovarian adenocarcinoma had stabilization of their disease, as assessed by palpation and CT scans, for 12 and 17 weeks, but there was evidence of progression at 17 and 21 weeks respectively.

Table II gives more detail of the 6 patients with testicular teratomas. There was X-ray evidence of tumour enlargement in all, and levels of either AFP, hCG or both markers rose in 5 patients whilst receiving etretinate. Several different histological types of malignant teratoma were treated, and at second-look operations after cytotoxic chemotherapy 3/6 showed evidence of tumour differentiation. However, only one of the 3 patients showing differentiation received etretinate before surgery and he (RR) had histological examination both before and after etretinate. Histology of the para-aortic mass removed after cytotoxic chemotherapy showed differentiated teratoma with some embryonal elements and rhabdomyoblasts. After 9 weeks of etretinate treatment alone, an enlarging mediastinal mass was removed, which showed differentiated teratoma with immature cartilage and rhabdomyoblasts.

Four patients with malignant teratoma (2 with melanoma, one with Hodgkin's disease and one with non-Hodgkin's lymphoma) received a total of 19 courses of cytotoxic chemotherapy whilst receiving etretinate (Table III). Marginal volume or tumour-marker responses were seen in 3 patients with teratomas. These responses were no greater than when the same combination (VP.16–213 + actinomycin D + cyclophosphamide; VP.16–213 + *cis*-platinum; cyclophosphamide + *cis*-platinum) had been given without etretinate, and were of very short duration. Similarly, the response to radiotherapy of one patient with melanoma, one with non-Hodgkin's lymphoma and one with teratoma whilst receiving etretinate was no greater than when given without. Etretinate did not increase the toxicity of radiotherapy or cytotoxic chemotherapy.

This study showed that in 17/19 evaluable patients, their tumours progressed whilst receiving retinoid therapy. The aromatic retinoid etretinate was chosen for this trial because oral administration produces signs of hypervitaminosis A without liver toxicity. In addition, it has a better therapeutic ratio than other retinoids tested in animal systems (Mayer *et al.*, 1978). Although in an *in vitro* system etretinate did not induce differentiation of

TABLE III.—*Etretinate with cytotoxic chemotherapy*

| Other agent | No. of patients | Single agent | In combination | Response |
|---|---|---|---|---|
| VP.16–213 | 5 | 7 | 6 | 3+ |
| *Cis*-platinum | 3 | 0 | 6 | 3+ |
| Cyclophosphamide | 2 | 0 | 2 | 2+ |
| Vindesine | 1 | 2 | 0 | – |
| Vinblastine | 1 | 1 | 0 | – |
| Actinomycin D | 1 | 0 | 1 | – |
| CCNU | 1 | 1 | 0 | – |
| Estramustine | 1 | 1 | 0 | – |

\+ Marginal.

embryonal carcinoma cells, its main acid metabolite was shown to be active, and there is a high serum concentration of this metabolite *in vivo* (Hanni *et al.*, 1979).

Etretinate did not induce differentiation of the malignant teratomas in the 6 evaluable patients with this tumour. There was histological evidence of undifferentiated active teratoma in one of these patients after 9 weeks' retinoid therapy. In the other 5 patients, in addition to enlargement of their tumour masses, there was a rise in their tumour markers whilst on etretinate.

One of the patients in whom there was no progression of tumour whilst on etretinate had metastatica melanoma. When melanoma metastasizes to the brain it almost invariably runs a progressive course. It is unlikely that this woman's tumour was eradicated by the cranial irradiation and CCNU, since the CT scan has remained abnormal throughout her treatment. Although it cannot be certain from one case that retinoid therapy may be beneficial in patients with metastatic melanoma, it is worth a trial in melanoma patients in view of its minimal toxicity and the absence of other successful treatment. The apparent temporary halt in disease progression in 2 patients with ovarian adenocarcinoma could be explained by the inadequacy of methods for monitoring this tumour.

Vitamin A was shown to enhance the antitumour effect of BCNU against murine L1210 leukaemia by Cohen & Carbone (1972) who suggested that this was related to their combined effects on membranes. No enhancement was shown in the present study, even with the lipid-soluble agents CCNU and VP.16–213.

Retinoids have many interesting properties, but this study suggests that much more experimental work is needed before a place can be found for them in human cancer chemotherapy.

Since this paper was prepared similar results have been reported in abstract form, using 13-*cis* retinoic acid in advanced cancer (Meyskens *et al.*, 1981).

We are grateful to Roche Products Ltd, Welwyn Garden City, Hertfordshire for the supply of etretinate, to Dr E. S. Newlands for allowing us to study his patients and to the Cancer Research Campaign for a Research Fellowship to G. J. S. Rustin.

REFERENCES

BOLLAG, W. & OTT, F. (1971) Therapy of actinic keratoses and basal cell carcinomas with local application of vitamin A acid (NSC-122758) *Cancer Chemother. Rep.*, **55**, 59.

COHEN, M. H. & CARBONE, P. P. (1972) Enhancement of the antitumour effects of 1, 3-bis (2-chloroethyl)-1-nitrosourea and cyclophosphamide by Vitamin A. *J. Natl Cancer Inst.*, **48**, 921.

HANNI, R., HERBOUTET, D. & BUSSLINGER, A. (1979) Determination of an aromatic retinoid and its main metabolite by high performance liquid chromatography. *J. Chromatogr.*, **162**, 615.

HONG, W. K., WITTES, R. E., HAJDU, S. T., CVITKOVIC, E., WHITMORE, W. F. & GOLBEY, R. B. (1977) The evolution of mature teratoma from malignant testicular tumours. *Cancer*, **40**, 2987.

JETTEN, A. M. & JETTEN, M. E. R. (1979) Possible role of retinoic acid binding protein in retinoid stimulation of embryonal carcinoma cell differentiation. *Nature*, **278**, 180.

KISTLER, G. S. & PETER, H. J. (1979) Wirkung von zwei Retinoiden (Vitamin-A-Analogen) auf menschliche Bronchus-karzinome *in vivo* (nu/nu/Maus) und *in vitro*. *Schweiz. Med. Wschr.*, **109**, 847.

LANCET (Editorial) (1981) Retinoids in dermatology. *Lancet* i, 537.

LEVINE, N. & MEYSKENS, F. L. (1980) Topical vitamin A acid therapy for cutaneous metastatic melanoma. *Lancet*, ii, 224.

LOTAN, R. (1980) Effects of vitamin A and its analogues (retinoids) on normal and neoplastic cells. *Biochim. Biohpys. Acta*, **605**, 33.

MAYER, H., BOLLAG, W., HANNI, R. & RUEGG, R. (1978) Retinoids, a new class of compounds with prophylactic and therapeutic activities in oncology and dermatology. *Experientia*, **34**, 1105.

MEYSKENS, F. L., JR, GILMARTIN, E., CHASE, E. & 4 others (1981) A broad phase II trial of 13-cis-retinoic acid in advanced cancer. *Proc. Am. Ass. Cancer Res. & ASCO*, **22**, 370.

MICKSCHE, M., CERNI, C., KOKRON, O., TITSCHER, R. & WERBA, H. (1977) Stimulation of immune response in lung cancer patients by vitamin A therapy. *Oncology*, **34**, 234.

PECK, G. L., OLSEN, T. G., BUTKUS, D. & 4 others (1979) Treatment of basal cell carcinoma with 13-cis-retinoic acid. *Proc. Am. Ass. Cancer Res. & ASCO*, **20**, 56.

TROWN, P. W., BUCK, M. J. & HANSEN, R. (1976) Inhibition of growth and regression of a transplantable rat chondrosarcoma by three retinoids. *Cancer Treat. Rep.*, **60**, 1647.